# Self-reported prenatal tobacco smoke exposure, *AXL* gene-body methylation, and childhood asthma phenotypes

**DOI:** 10.1186/s13148-018-0532-x

**Published:** 2018-07-20

**Authors:** Lu Gao, Xiaochen Liu, Joshua Millstein, Kimberly D. Siegmund, Louis Dubeau, Rachel L. Maguire, Junfeng (Jim) Zhang, Bernard F. Fuemmeler, Scott H. Kollins, Cathrine Hoyo, Susan K. Murphy, Carrie V. Breton

**Affiliations:** 1 Department of Preventive Medicine, USC Keck School of Medicine, 2001 N. Soto Street, Los Angeles, CA 90032 USA; 2 Department of Biological Sciences, Center for Human Health and the Environment, North Carolina State University, Raleigh, NC 27695 USA; 3 Nicholas School of the Environment and Duke Global Health Institute, Duke University, Durham, NC 27701 USA; 4 Department of Health Behavior and Policy, Massey Cancer Center, Virginia Commonwealth University, Richmond, VA 23219 USA; 5 Department of Psychiatry and Behavioral Sciences, Duke University Medical Center, Durham, NC 27705 USA; 6 Division of Reproductive Sciences, Department of Obstetrics and Gynecology, Duke University School of Medicine, Durham, NC 27708 USA

**Keywords:** Methylation, Epigenetics, Smoke

## Abstract

**Background:**

Epigenetic modifications, including DNA methylation, act as one potential mechanism underlying the detrimental effects associated with prenatal tobacco smoke (PTS) exposure. Methylation in a gene called *AXL* was previously reported to differ in response to PTS.

**Methods:**

We investigated the association between PTS and epigenetic changes in *AXL* and how this was related to childhood asthma phenotypes. We tested the association between PTS and DNA methylation at multiple CpG loci of *AXL* at birth using Pyrosequencing in two separate study populations, the Children’s Health Study (CHS, n = 799) and the Newborn Epigenetic Study (NEST, n = 592). Plasma cotinine concentration was used to validate findings with self-reported smoking status. The inter-relationships among *AXL* mRNA and miR-199a1 expression, PTS, and *AXL* methylation were examined. Lastly, we evaluated the joint effects of *AXL* methylation and PTS on the risk of asthma and related symptoms at age 10 years old.

**Results:**

PTS was associated with higher methylation level in the *AXL* gene body in both CHS and NEST subjects. In the pooled analysis, exposed subjects had a 0.51% higher methylation level in this region compared to unexposed subjects (95% CI 0.29, 0.74; *p* < 0.0001). PTS was also associated with 21.2% lower expression of miR-199a1 (95% CI − 37.9, − 0.1; *p* = 0.05), a microRNA known to regulate *AXL* expression. Furthermore, the combination of higher *AXL* methylation and PTS exposure at birth increased the risk of recent episodes of bronchitic symptoms in childhood.

**Conclusions:**

PTS was associated with methylation level of *AXL* and the combination altered the risk of childhood bronchitic symptoms.

**Electronic supplementary material:**

The online version of this article (10.1186/s13148-018-0532-x) contains supplementary material, which is available to authorized users.

## Background

Tobacco smoking during pregnancy has been linked to several perinatal complications and child health problems [1]. Previous studies have associated prenatal tobacco smoke (PTS) exposure with low birth weight [2], preterm delivery [3], increased asthmatic symptoms, and reduced pulmonary function in childhood [4, 5], as well as cancer in adult life [6]. One hypothesized mechanism for the adverse health effects of PTS on offspring is through epigenetic modifications such as DNA methylation and microRNA expression [7–9]. Alterations in the epigenome established in utero may last across the child’s life course to affect disease phenotypes much later and may influence gene expression at various developmental stages. A deeper understanding of the complexities underlying epigenetic responses to PTS, and how these epigenetic changes may affect the health and behavior of offspring, is still lacking.

We previously reported that DNA methylation in the promoter region of *AXL*, a receptor tyrosine kinase of the TAM (*TYRO3*, *AXL*, and *MERTK*) family, was susceptible to maternal smoking during pregnancy [10, 11]. *AXL* was originally discovered in cancer cells and has been shown to regulate various functions including cell survival and growth, clearance of apoptotic cells, and natural killer cell differentiation [12–14]. More recently, studies have shown that *AXL* and its major ligand growth-arrest-specific 6 (*GAS6*) also play an anti-inflammation role by limiting the production of Toll-like receptor (TLR)-induced proinflammatory cytokines [15]. Childhood asthma is the most common chronic disease among children and largely involves airway inflammation [16, 17]. *GAS6* showed higher expression in subjects with severe asthma during exacerbation [18]. However, few studies have addressed the epigenetic regulation of *AXL* in the pathogenesis of childhood asthma [19]. The role that PTS may play in modifying these processes is similarly unknown.

Our previous research in the Children’s Health Study (CHS) demonstrated that PTS exposure was associated with increased *AXL* DNA methylation in childhood of one CpG locus located in an Sp1/Sp3 transcription factor binding region, at which the methylation level was reported to correlate with gene expression of *AXL* [10, 20]. In addition to regulation by CpG methylation in its promoter directly, *AXL* expression is also negatively regulated by microRNA 199a1 (miR-199a1) [21]. Based on the above evidence, we sought to investigate the effects of PTS exposure on *AXL* methylation, mRNA expression, and miR-199a1 expression in the offspring earlier in life, at the time of birth. We further hypothesized that *AXL* methylation would be associated with later childhood asthma and related symptoms through innate immune pathways involved in the pathogenesis of asthma.

In this study, we first tested the association between PTS exposure and DNA methylation at multiple CpG loci across the regulatory regions of *AXL* using Pyrosequencing in two separate study populations, the Children’s Health Study (CHS) [22] and the Newborn Epigenetic Study (NEST) [23]. In NEST, plasma cotinine concentration was also used to validate our findings using self-reported PTS. We then evaluated the inter-relationships among *AXL* mRNA and miR-199a1 expression, PTS exposure, and *AXL* CpG methylation. Lastly, for functional follow-up of our findings, we evaluated the joint effects of *AXL* methylation and PTS exposure by testing their interaction on the risk of asthma and related symptoms at the age of 10 years in the CHS.

## Methods

### Study population

This study was primarily conducted in subsets of participants of the Children’s Health Study (CHS), a longitudinal study of respiratory health of children in southern California [22, 24–26]. Any subjects with reported chest surgery, chest injury, or cystic fibrosis were excluded from the study population. Based on our ability to link CHS subjects with California birth records and to obtain a newborn bloodspot, a subset of 799 children was selected for an epigenetic study in which DNA methylation at multiple CpG loci on *AXL* was assessed using Pyrosequencing. The sample selected was enriched with subjects exposed to PTS.

Participant’s health, personal, parental, socio-demographic characteristics, and medical history were obtained from parent-completed questionnaires at study enrollment and were updated annually throughout the study. Children were considered to have a history of asthma if there was a yes answer the question “Has a doctor ever diagnosed this child as having asthma?” History of wheezing was defined by a yes answer to the question “Has your child’s chest ever sounded wheezy or whistling?” and the same question was asked to evaluate wheezing in the previous 12 months. Bronchitic symptoms during the previous 12 months was defined based on the parent’s report of a daily cough for 3 months in a row, congestion or phlegm other than when accompanied by a cold or bronchitis.

A subset of 592 Newborn Epigenetic Study (NEST) subjects was also evaluated for the association between PTS exposure and *AXL* methylation. NEST is a prospective study of women and their children [23]. It was designed to identify exposures during pregnancy and early life associated with stable epigenetic alterations in infants that may alter chronic disease susceptibility later in life. Women were recruited from prenatal clinics serving Duke University Hospital and Durham Regional Hospital Obstetrics facilities in Durham, North Carolina and were eligible if they were aged 18 years and older, pregnant, and spoke English or Spanish. Participating women were either consented and interviewed in-person or were given the questionnaire to self-administer and mail back to the study office. Smokers were preferentially enrolled to the extent possible, identified through medical records. The NEST subjects are still under active follow-up and are too young to be assessed for asthma and related symptoms in childhood.

### Prenatal tobacco smoke exposure assessment

In CHS participants, a child was considered to be exposed to PTS if the parent completing the questionnaire answered yes to the question “Did this child’s biologic mother smoke while she was pregnant with this child?” and unexposed if the answer was no. NEST participants were considered to be exposed to PTS if the mother reported having ever smoked 100 cigarettes or more in her lifetime and smoking at any time during the pregnancy. NEST subjects were classified as unexposed if the mother reported never having smoked 100 cigarettes or more in her lifetime, or if the mother reported currently not smoking and not smoking anytime in the year before she knew she was pregnant.

### Cotinine collection and assay procedures in NEST

Maternal tobacco smoking in NEST was also evaluated by measuring cotinine concentration in maternal plasma samples taken during pregnancy at the time of initial recruitment into the study. Plasma blood samples were collected from women during pregnancy. Assays were completed at the Exposure Biology and Chemistry Lab at Duke University. Cotinine was measured using a high-performance liquid chromatography with tandem mass spectrometric detection (HPLC-MS-MS) method, a highly sensitive assay designed to measure levels of environmental smoke exposure with a limit of detection of 0.05 ng/ml [27–29] and a reproducibility > 94% [27–29]. Details of cotinine assay procedures were described elsewhere [30].

### DNA methylation

For CHS subjects, DNA methylation was measured in newborn bloodspots (NBS) that were obtained as part of the routine California Newborn Screening Program from the California Department of Public Health Genetic Disease Screening Program. The NBS were stored by the state of California at − 20 °C. A single complete newborn bloodspot for each requested participant was mailed to us and then stored in our lab at − 80 °C upon receipt. DNA was extracted from whole blood cells using the QiaAmp DNA blood kit (Qiagen Inc., Valencia, CA) and stored at − 80 °C. For NEST subjects, genomic DNA from buffy coat specimens was extracted from umbilical cord blood using Puregene Reagents (Qiagen, Valencia, CA). Laboratory personnel performed DNA methylation analysis by Pyrosequencing (PSQ) [31] and were blinded to study subject information.

**Fig. 1:**
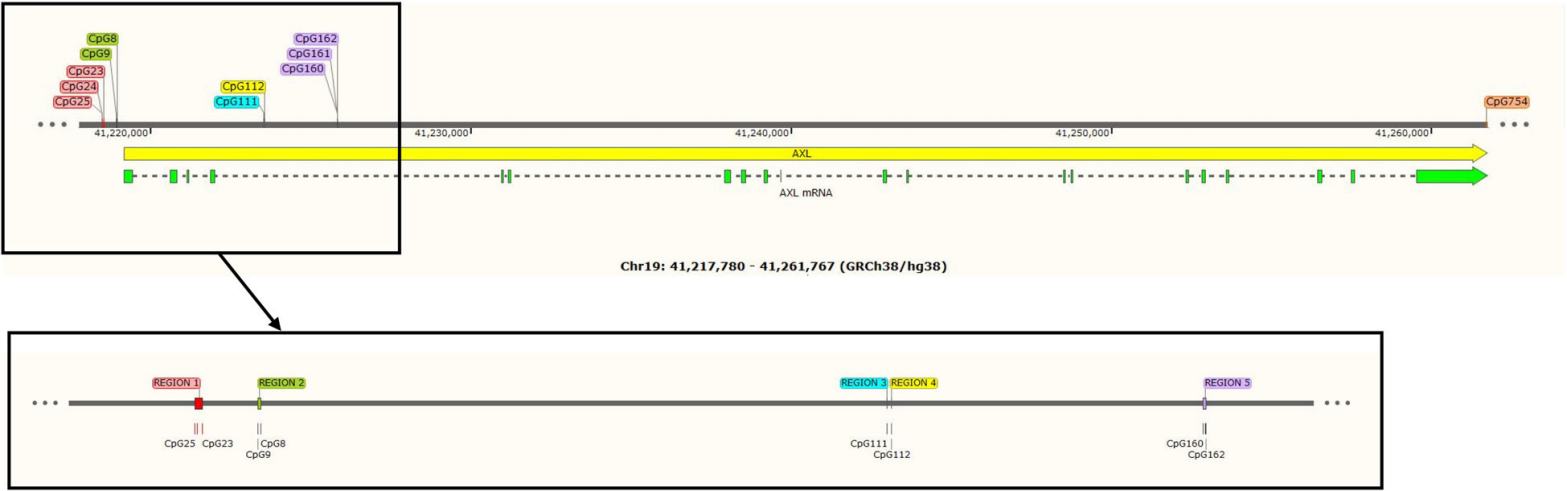
Genomic locations of *AXL* CpG sites under investigation. CpG sites captured by the same primer set are labeled in the same color to show categorization of the CpG sites into 6 regions. CpG 25 was previously reported to associate with prenatal tobacco smoke exposure (reference #10). CpGs 23, 9, 112, 161 and 754 were previously studied in relation to childhood asthma and related symptoms (reference #19)

Six regions across *AXL* were selected for PSQ, each containing one to three CpGs (Fig. 1). PCR primers were designed by EpigenDx Inc. (http://www.epigendx.com) to cover the loci of interest and the specificity of the primer sequences was confirmed using in silico PCR. Five hundred nanograms of genomic DNA extracted from NEST and CHS samples (randomized together) was bisulfite treated using the EZ DNA Methylation Kit™ (Zymo Research, Irvine, CA, USA) and was purified according to the manufacturer’s protocol. Methylation assays (assays ADS6525-FS, ADS8094-FS2, ADS6528-FS, ADS8097-FS, and ADS6570-FS) were performed by EpigenDx Inc. using the PSQ96HS system (Pyrosequencing, Qiagen) according to standard procedures as described in previous work [32, 33]. Two percent of the samples were measured in duplicate to evaluate reproducibility. The methylation level was determined using QCpG software (Pyrosequencing, Qiagen) and was reported as percent of DNA methylation at each CpG locus. Each experiment included cytosines not part of a CpG dinucleotide as internal controls to evaluate incomplete bisulfite conversion of the input DNA. A series of unmethylated and methylated DNA samples were included as controls in each assay. Furthermore, PCR bias testing was performed by mixing unmethylated control DNA with in vitro methylated DNA at different ratios (0, 5, 10, 25, 50, 75, and 100%), followed by bisulfite modification, PCR, and Pyrosequencing analysis. Standard curves for pre-specified ratios against the measured methylation levels were then produced for each assay to check PCR bias and were shown in Additional file 1: Figure S1.

### miRNA and mRNA expression in NEST

All measurements of miR-199a1 and *AXL* mRNA expression were conducted in duplicate in cord blood samples from 235 participants in the NEST cohort. Total mRNA was isolated from stored PAXgene tubes of cord blood using the PAXgene blood miRNA isolation kit (Qiagen, Valencia, CA). Expression of miR-199a1 was quantified using Origene’s qStar miRNA detection system (Origene, Rockville, MD) with qStar primer pairs specifically designed for the target (miR-199a1 transcript #HP300226) and its corresponding copy number standard (#HK300226). Plasmid DNA containing a cloned fragment of the target gene was used as the qPCR copy number standard. By utilizing this qPCR standard for the miRNA and employing the standard curve qPCR method, we calculated the absolute copy number of miR-199a1 in each sample. To evaluate reproducibility, 10% repeats were included. Additional details of mRNA expression measurements are described in the Additional file 1: Supplemental methods.

### Statistical methods

We first conducted descriptive analyses to examine the distribution of CpG methylation, miR-199a1 and *AXL* mRNA expression, and the population characteristics of both CHS and NEST study participants.

**Fig. 2:**
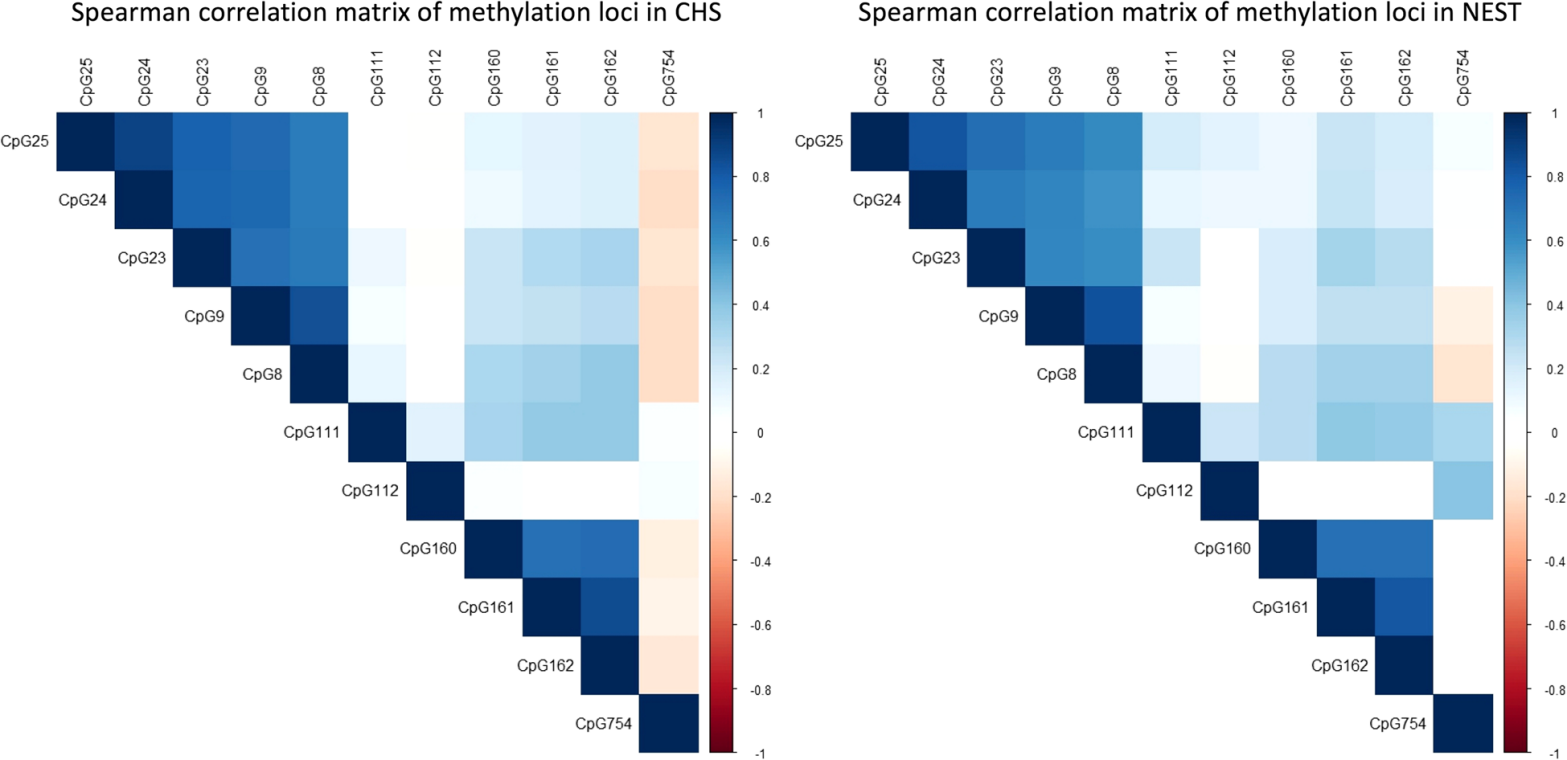
Spearman correlation between methylation at each *AXL* CpG site under investigation in CHS (left) and NEST (right) subjects. Positive correlations are displayed in blue and negative correlations in red color. Color intensity is proportional to the correlation coefficients

Spearman correlations between methylation levels at CpG sites under investigation were evaluated (Fig. 2). Except for CpG 111 and CpG 112, the methylation levels at CpG sites captured by the same primer set were generally highly correlated, so we averaged their methylation values. In total, six regions across *AXL* were defined (Fig. 1): region 1 was the average of CpG 23-25; region 2 was CpG 8-9; region 3 was CpG 111; region 4 was CpG 112; region 5 was CpG 160-162; and region 6 was CpG 754.

To estimate the main effects of PTS exposure on *AXL* methylation, we first fitted linear regression models using the dichotomized PTS from self-reported questionnaire as described above, and adjusted for child’s sex, ethnicity (of child in CHS and of mother in NEST), gestational age, maternal age at delivery, and parental education level. Separate analyses for CHS and NEST were conducted first. Then, a pooled analysis was also conducted, but only included Hispanic, non-Hispanic white, and black subjects due to the different ethnic distribution of these two populations, leaving 1294 subjects for analysis (725 CHS subjects and 569 NEST subjects). All potential covariates were chosen for inclusion based on a priori hypotheses. DNA methylation plate number, parity, and *AXL* genetic polymorphisms did not change the effect estimates by more than 10% and were removed from final models.

In addition to main effects of PTS, we also stratified the analyses by child’s sex and ethnicity and tested for interaction by adding the corresponding interaction terms into the models described above. Additionally, to evaluate the association between maternal plasma cotinine concentration and *AXL* methylation in the NEST population, we first categorized cotinine level as nonsmoker (0–1 ng/ml), passive smoker (1–10 ng/ml), and active smoker (> 10 ng/ml), and fitted linear regression models with *AXL* methylation [34, 35]. To further explore the nonlinearity of this relationship, we also fitted a linear regression model of log10-transformed cotinine and *AXL* methylation with the two-component linear piecewise spline with a break at 10 ng/ml peak exposure:

$$ \mathrm{Methylation}=\upalpha +{\beta}_1\times \mathrm{cotinine}+{\beta}_2\times {\left(\mathrm{cotinine}-10\right)}^{+}+\mathrm{covariates}+\varepsilon . $$

where (cotinine − 10)^+^ takes on a value of 0 when cotinine level is smaller than 10 ng/ml. These models were adjusted for the same covariates as in models using self-reported PTS as well as maternal BMI during pregnancy.

We also evaluated the association between PTS and the expression levels of *AXL* mRNA and miR-199a1 in the NEST population by fitting individual linear regression models. The models were adjusted for child’s sex, maternal ethnicity, gestational age, maternal age at delivery, maternal education level, maternal BMI during pregnancy, and miR-199a1 assay number (for miR-199a1 models). Similar models were fitted to assess the association between *AXL* methylation and the expression of *AXL* mRNA and miR-199a1.

Lastly, to evaluate the joint effects of *AXL* methylation and maternal smoking on childhood asthma and related symptoms at age 10 years in CHS subjects, we fitted logistic regression models. An interaction term between methylation and PTS was included, adjusted for child’s sex, ethnicity, and city of residence at study entry. Wald tests were used to compute interaction *p* values.

Statistical analyses were performed using SAS (Statistical Analysis System) version 9.4 (SAS Institute, Cary, NC) and R version 3.3.1 software.

## Results

The population characteristics of the CHS and NEST study subjects are described in Table 1. Children’s sex was evenly distributed in both populations, while the distribution of ethnicity differed between the two by design. Of the 799 CHS participants, 39% of them were Hispanic white, 49% were non-Hispanic white, and 12% were a mixture of Asian, black, and others; whereas in the 592 NEST participants, there were a much higher proportion of subjects born to black mothers (51%). Both studies were enriched for participants exposed to PTS, and a higher proportion of exposed subjects were observed in NEST (51%) than CHS (27%). With the current sample, 18% of CHS subjects had a history of physician-diagnosed asthma by age 10 years, and more subjects had experienced wheezing symptoms. Less than 20% of CHS subjects had wheezing or bronchitic symptoms in the previous 12 months.

**Table 1:** Demographic characteristics of CHS and NEST subjects

	CHS (*N* = 799)	NEST (*N* = 592)	Pooled analysis (*N* = 1294)^a^
	*n* (%)	*n* (%)	*n* (%)
Sex			
Male	366 (45.8%)	311 (52.5%)	632 (48.8%)
Female	433 (54.2%)	281 (47.5%)	662 (51.2%)
Ethnicity^b^			
Asian	29 (3.6%)	–	–
Black	23 (2.9%)	299 (50.5%)	322 (24.9%)
Hispanic	308 (38.6%)	58 (9.8%)	366 (28.3%)
Non-Hispanic white	394 (49.3%)	212 (35.8%)	606 (46.8%)
Other	45 (5.6%)	23 (3.9%)	–
Highest parental education level^c^			
Less than 12th grade	102 (12.8%)	126 (21.3%)	222 (17.2%)
Completed grade 12	153 (19.2%)	162 (27.4%)	298 (23.0%)
Some college or tech school	352 (44.1%)	159 (26.9%)	483 (37.3%)
Completed 4 years of college or higher	175 (21.9%)	142 (24.0%)	208 (16.1%)
Maternal smoking during pregnancy	217 (27.2%)	303 (51.2%)	500 (38.6%)
Ever MD-diagnosed asthma^d^	141 (17.7%)	–	–
Ever wheezing^d^	284 (35.5%)	–	–
Wheezing in the previous 12 months^d^	145 (18.2%)	–	–
Bronchitic symptoms in the previous 12 months^d^	118 (14.8%)	–	–
Gestational age (weeks), mean ± SD	39.6 ± 2.0	38.6 ± 2.2	39.2 ± 2.1
Maternal age at delivery (years), mean ± SD	27.8 ± 5.9	27.7 ± 5.8	27.7 ± 5.8

Percent number do not always add up to 100% due to missing data

^a^Pooled analysis only included black, Hispanic and non-Hispanic white subjects

^b^Ethnicity of child in CHS and of mother in NEST

^c^Highest education level of either parent in CHS and of mother in NEST

^d^Assessed at mean age 9.96 years (SD 0.37)

The distribution of *AXL* methylation in each region under investigation was similar across NEST and CHS subjects (Additional file 1: Table S1). Region 4 was highly methylated with a mean value of 84% (SD = 3.1%) in CHS, and 83% (SD = 5.3%) in NEST. Regions 1, 3, and 6 were moderately methylated with mean values ranging from 37 to 58%. CpG loci in regions 2 and 5 were mostly unmethylated, with mean values ranging from 4 to 13%. miR-199a1 expression level was heavily right-skewed with a median of 184,098 copies/μg RNA (IQR = 258,974 copies/μg RNA) (Additional file 1: Table S1). mRNA expression in cord blood was relatively low and right-skewed (median = 3503 copies/μg cDNA, IQR = 1965 copies/μg cDNA). Cotinine values were also right-skewed, with a median of 1.0 ng/ml (IQR = 28 ng/ml).

### Main effects of PTS

We evaluated the association between self-reported PTS exposure and *AXL* methylation in both CHS and NEST populations (Table 2). In CHS, methylation level in region 5 at birth was significantly higher in participants exposed to PTS compared to unexposed participants (*β* = 0.56; 95% CI 0.31, 0.82; *p* < 0.0001). We found a similar association at this region in NEST (*β* = 0.35; 95% CI − 0.03, 0.73; *p* = 0.07) and in the pooled analysis (*β* = 0.51; 95% CI 0.29, 0.74; *p* < 0.0001). In addition, participants exposed to PTS had a 0.58% higher methylation in region 3 compared to the unexposed in CHS subjects, but this association was not replicated in NEST. No associations were observed between PTS and methylation in the other regions. Results for the individual CpG sites in each region were also presented in Additional file 1: Table S2 and were quite consistent with the regional averages. We also tested whether these associations varied by child’s sex or ethnicity, but found no significant differences (results not shown).

**Table 2:** Association between PTS exposure and *AXL* DNA methylation at birth in CHS and NEST subjects (adjusted for child’s sex, ethnicity (of child in CHS and of mother in NEST), gestational age, maternal age at delivery, and parental education level)

	CHS (*N* = 799)		NEST (*N* = 592)		Pooled analysis (*N* = 1294)^a^	
	훽β (95% CI)	*P* value	β (95% CI)	*P* value	β (95% CI)	*P* value
Region 1	− 0.49 (− 1.56, 0.58)	0.37	0.11 (− 1.16, 1.37)	0.87	− 0.30 (− 1.11, 0.51)	0.47
Region 2	0.01 (− 0.57, 0.59)	0.97	0.59 (− 0.23, 1.42)	0.16	0.23 (− 0.25, 0.72)	0.34
Region 3	0.58 (− 0.06, 1.22)	0.08	− 0.22 (− 1.48, 1.04)	0.73	0.24 (− 0.40, 0.88)	0.47
Region 4	− 0.12 (− 0.67, 0.43)	0.67	0.55 (− 0.45, 1.54)	0.28	0.14 (− 0.41, 0.69)	0.61
Region 5	0.56 (0.31, 0.82)	1.72E−05	0.35 (− 0.03, 0.73)	0.07	0.51 (0.29, 0.74)	7.89E−06
Region 6	0.46 (− 0.16, 1.09)	0.14	− 0.53 (− 1.88, 0.81)	0.44	− 0.10 (− 0.78, 0.57)	0.77

Estimates are showing percent changes in methylation

^a^Pooled analysis only included black, Hispanic, and non-Hispanic white subjects

We further evaluated the association between maternal smoking and *AXL* methylation in NEST subjects using plasma cotinine concentration categorized as nonsmoker (0–1 ng/ml), passive smoker (1–10 ng/ml), and active smoker (> 10 ng/ml) (Additional file 1: Table S3). Methylation levels in regions 1, 3, 4, and 5 were positively associated with cotinine level, but none of the effect estimates reached statistical significance, possibly due to a small sample size. To further explore the nonlinear dose-response relationship of cotinine and methylation, we used a piecewise linear regression model with a break at 10 ng/ml and found that methylation levels in regions 1 (*p* = 0.0001), 2 (*p* = 0.001), and 6 (*p* = 0.002) were significantly associated with cotinine level (Additional file 1: Figure S3).

### Association between miR-199a1 and AXL mRNA with PTS and AXL methylation

We measured the expression of both *AXL* mRNA and miR-199a1, a microRNA known to regulate *AXL* gene expression, and tested their associations with PTS and *AXL* methylation (Additional file 1: Table S4 and Table S5). We found that *AXL* mRNA was expressed at low levels in cord blood, which is consistent with the relatively low expression in whole blood compared to other tissues using data from the Genotype-Tissue expression (GTEx) project (Additional file 1: Figure S3). In our population, *AXL* mRNA was not associated with self-reported PTS, cotinine concentration, or *AXL* methylation, but was marginally and negatively associated with methylation in region 4 (*p* = 0.07) and miR-199a1 levels (*p* = 0.10) (Additional file 1: Table S4).

However, miR-199a1 was associated with methylation and PTS. A 1% higher level of methylation in region 4 was significantly associated with a 2.1% increase in miR-199a1 expression level (95% CI 0.7, 3.5; *p* = 0.05) (Additional file 1: Table S5). Subjects with PTS exposure had a 21.2% lower miR-199a1 expression level compared to unexposed subjects (95% CI − 37.9, − 0.1; *p* = 0.05). A similar association was observed using plasma cotinine level, for which a 10% increase in cotinine concentration was significantly associated with a 0.8% lower miR-199a1 level (95% CI − 1.3, − 0.2; *p* = 0.01).

### Interaction between PTS and AXL methylation on asthma and related symptoms

Given the previous findings that PTS was associated with increased risk of childhood asthma and related symptoms, and our observed associations between PTS and methylation in *AXL*, we next sought to test whether they might jointly interact to alter susceptibility to childhood asthma and related symptoms (Table 3). The association between methylation in region 5 and the risk of recent bronchitic symptoms at the age of 10 years significantly differed for subjects with and without PTS exposure (*p*-interaction = 0.01). Higher methylation level in region 5 at birth was associated with higher risk of bronchitic symptoms (OR = 2.26 per 2SD change in methylation; 95% CI 1.09, 4.72; *p* = 0.03) in children who were exposed to PTS, but not in unexposed children (OR = 0.76 per 2SD change in methylation; 95% CI 0.45, 1.29; *p* = 0.31). No significant interaction with PTS was observed for methylation in other regions on the risk of asthma and related symptoms (Table 3 and Additional file 1: Table S6).

**Table 3:** Interaction between *AXL* DNA methylation at birth and PTS exposure in relation to risk of bronchitic symptoms at age 10 years in CHS subjects (*N* = 799) (adjusted for child’s sex, ethnicity, and city of residence at study entry. Odds ratios are scaled to per 2SD change in methylation)

	Unexposed to PTS		Exposed to PTS		Interaction *P* value
	OR (95% CI)	*P* value	OR (95% CI)	*P* value	
Region 1	0.96 (0.59, 1.57)	0.87	1.52 (0.72, 3.20)	0.28	0.46
Region 2	1.21 (0.75, 1.96)	0.43	1.85 (0.87, 3.96)	0.11	0.53
Region 3	1.10 (0.69, 1.76)	0.68	1.35 (0.64, 2.82)	0.43	0.64
Region 4	0.93 (0.58, 1.47)	0.74	0.85 (0.40, 1.81)	0.67	0.90
Region 5	0.76 (0.45, 1.29)	0.31	2.26 (1.09, 4.72)	0.03	0.01
Region 6	0.91 (0.59, 1.40)	0.67	0.99 (0.46, 2.17)	0.99	0.62

## Discussion

We assessed the association between maternal smoking during pregnancy, epigenetic regulation of *AXL* and bronchitic symptoms in childhood. Consistent associations between self-reported PTS and higher *AXL* methylation in one region were observed in both CHS and NEST, despite different underlying population characteristics. Both self-reported PTS and plasma cotinine levels were associated with the expression of miR-199a1, a microRNA known to regulate *AXL* expression. Furthermore, we found synergistic effects between PTS and *AXL* methylation at birth on the risk of bronchitic symptoms 10 years later in childhood.

*AXL* methylation in the gene body (region 5) was significantly associated with self-reported PTS in CHS and the results were replicated in NEST. Results were unaffected by differences in ethnic distributions or by the use of newborn bloodspots versus cord blood in two populations. However, we previously reported PTS was associated with *AXL* methylation level at CpG 25 in region 1 in a separate subset of CHS subjects [10, 11] but could not replicate this association in the current study. Discrepancies between these findings may be due to a number of factors including differences in cell type and timing of exposure. For example, *AXL* methylation was assessed in buccal cells in the previous papers but in newborn bloodspots in this paper; differences may therefore be due to differences in tissue and cell type [36]. More importantly, *AXL* methylation was measured in childhood in previous studies, but was assessed at birth in this paper, which is a more relevant time window with respect to in utero smoke exposure. Given the dynamic and time-specific nature of DNA methylation, the previously observed changes in region 1 may reflect not only exposure from PTS, but also a summary of other postnatal and childhood exposures such as air pollution, diet, exercise, and environmental tobacco smoke exposure.

Because self-reported PTS may introduce measurement error, we also tested the effects of plasma cotinine level, which is currently regarded as the best biomarker of smoke inhalation in active smokers and in nonsmokers exposed to environmental tobacco smoke [37]. We explored both the linear (results not shown) and non-linear relationship of maternal plasma cotinine level and *AXL* methylation in each region. The results were supportive of our findings using self-reported PTS, with the same direction of associations for region 5, and might suggest a non-linear dose-response relationship, though power was limited.

**Fig. 3:**
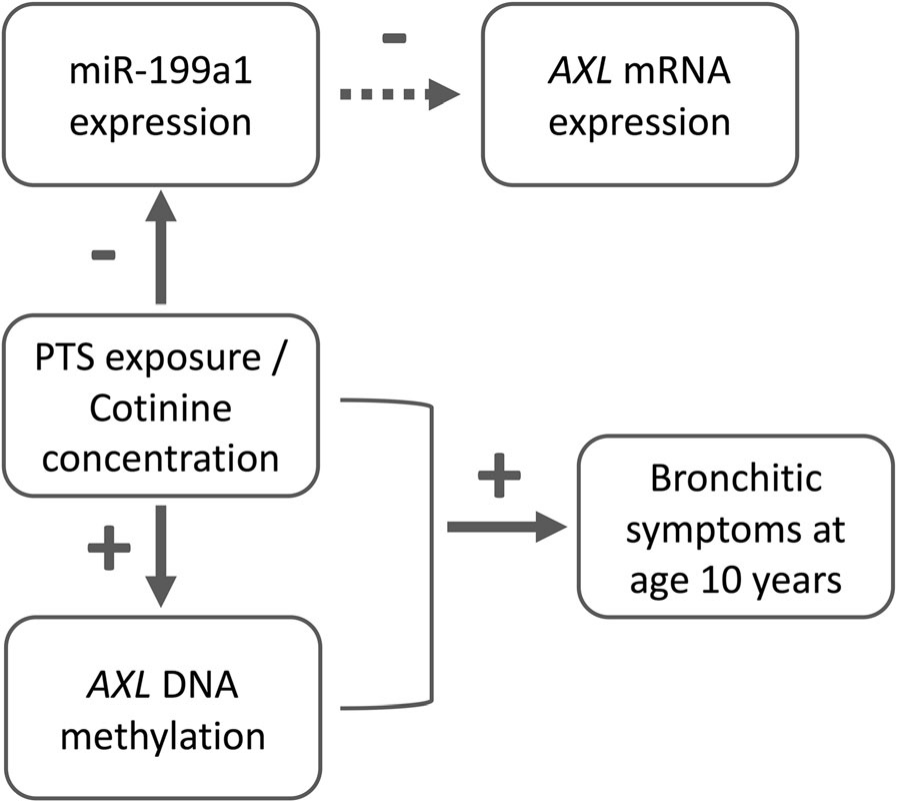
Illustration of the association between PTS, epigenetic regulation of *AXL*, and bronchitic symptoms in childhood

In addition to DNA methylation, both PTS and cotinine level were negatively associated with miR-199a1 expression in this study. Given the generally low expression of *AXL* in cord blood, we were not able to convincingly relate PTS to *AXL* mRNA level. Nonetheless, miR-199a1 expression was marginally associated with lower *AXL* mRNA expression, which is consistent with the previously reported role of miR-199a1 to negatively regulate *AXL* expression in cancer cell lines [21]. Taken together, these findings illustrate the effects of PTS on multiple aspects of *AXL* regulation (Fig. 3), including methylation at multiple loci, microRNA expression, and potentially mRNA expression.

PTS has been associated with impaired growth in fetal brain, lung, and kidney and infant morbidity and mortality [38–40], lifelong decreases in pulmonary function, and increased risk of childhood asthma [41, 42]. Epigenetic programming may be involved in the consequences of PTS on offspring health outcomes. PTS is now clearly linked to alterations in newborn DNA methylation patterns in multiple tissues, which may persist into childhood and even adolescence and are further linked to adverse fetal and childhood health outcomes [9, 43–46]. Given *AXL*’s key role in innate immune inflammation and lung homeostasis pathways [13–15, 47], it stands to reason that a systemic alteration to *AXL* regulation during fetal development by an exposure such as tobacco smoke might negatively impact function of all tissues and cells in which *AXL* has an active role.

We aimed to investigate this hypothesis—of altered *AXL* programming increasing downstream vulnerability—by investigating whether *AXL* methylation at birth was related to childhood respiratory health. To do so, we tested the interaction between PTS and *AXL* methylation on the risk of childhood asthma and related phenotypes. Methylation in region 5, which showed the strongest association with PTS, was associated with increased risk for recent episodes of bronchitic symptoms only in subjects exposed to PTS. No interactions were observed for asthma or wheezing outcomes. Bronchitic symptoms are suggestive of chronic symptoms that may follow an illness or acute exacerbation of asthma, or chronic inflammation in the airway. The observed synergistic effects of PTS exposure and *AXL* methylation for higher risk of bronchitic symptoms still held after adjusting for asthma status (results not shown). Interaction between smoke exposure and gene methylation has also been reported to alter the risk of other respiratory diseases [48]. Therefore, one would postulate that PTS exposure may enhance the influence of *AXL* methylation on innate immune pathways and further modify the risk of developing inflammatory phenotypes.

The current study has several strengths. The assessment of methylation pattern occurred in a relevant exposure time window. The prenatal period is highly sensitive to environmental factors and particularly susceptible to epigenetic alterations due to rapid cell division and epigenetic remodeling [49]. Thus, alterations in methylation status at birth may serve as a biomarker for direct consequences of environmental toxicants. We also examined the association between PTS and *AXL* DNA methylation in two separate populations of subjects and were able to replicate the significant findings despite differences in population demographics. Additionally, plasma cotinine concentration was used to validate results with self-report of maternal smoking. Lastly, we related DNA methylation levels at birth to childhood respiratory symptoms 10 years later, removing the possibility of reverse causation.

Several limitations should also be noted. First, our observed effect estimates in DNA methylation associated with PTS are small. However, most studies on DNA methylation changes in newborns in relation to maternal smoking during pregnancy, including meta-analysis of several large cohorts, all identified changes with small magnitudes as low as or less than 0.5% [9, 50–52]. A previous study defined the mean methylation change in response to PTS for both hyper- and hypomethylation as 2% [51]. Furthermore, we previously published a review and summarized that most environmental exposure studies have reported changes in DNA methylation from 2 to 10%, highlighting possible reasons for such small effects and the call for focusing on small magnitude alterations [53]. Second, although we evaluated the effects of PTS on multiple aspects of *AXL* epigenetic modifications, we were not able to test the circulating protein level of *AXL* to functionally interpret the changes in epigenetic marks. Future animal studies may elucidate this by measuring *AXL* protein level in relation to smoke exposure and determining whether this correlates with *AXL* methylation and mRNA levels. Third, PTS was only classified as a dichotomous variable in the analysis, raising the possibility that we may miss finer resolutions in exposure assessment, such as trimester-specific smoking effects. We were also unable to test whether the DNA methylation changes observed at birth persisted into childhood, as the CHS does not have childhood blood samples. Besides, asthma and related symptoms in childhood were self-reported in questionnaire and may introduce misclassification bias, however, a previous CHS study has independently verified self-reported physician diagnosed-asthma through a review of medical records and found that the information was highly reliable [54]. Lastly, although we made every effort to control for potential confounders, the possibility of residual confounding by some unknown factors associated with *AXL* DNA methylation levels and PTS cannot be ruled out, for instance, cell type compositions in blood and diet. Future studies may address these issues by inferring cell type compositions from Pyrosequencing methylation data of large numbers of genes.

## Conclusions

In conclusion, prenatal tobacco smoke exposure was associated with the regulation of *AXL,* a receptor tyrosine kinase of the TAM family that plays an important role in suppressing Toll-like receptor-induced inflammation. Moreover, *AXL* methylation and tobacco smoke exposure during fetal development may act synergistically to modify the risk of bronchitic symptoms in childhood.

## Additional file

Additional file 1: Supplemental methods. **Table S1.** Summary statistics for miR-199a1 and *AXL* mRNA expression, cotinine concentration and AXL methylation levels in CHS and NEST subjects. **Table S2.** Association between PTS exposure and AXL DNA methylation at each CpG site at birth in CHS and NEST subjects. **Table S3.** Association between plasma cotinine level and *AXL* methylation in NEST subjects. **Table S4.** Association between *AXL* mRNA expression and *AXL* methylation, PTS exposure and miR-199a1 expression in NEST subjects. **Table S5.** Association between miR-199a1 expression and *AXL* methylation and PTS exposure in NEST subjects. Table S6. Interaction between *AXL* DNA methylation at birth and PTS exposure in relation to risk of asthma and related symptoms at age 10 years in CHS subjects. **Figure S1.** Standard curve for testing PCR bias in Pyrosequencing assays. **Figure S2.** Plots for association between plasma cotinine level and *AXL* methylation using piecewise linear regression model. **Figure S3.** *AXL* mRNA expression levels across different tissues from the Genotype-Tissue expression (GTEx) project. (DOCX 790 kb)

## Abbreviations

BMI: Body mass index
CHS: Children’s Health Study
CI: Confidence interval
CpG: Cytosine-guanine dinucleotide sites
GTEx: Genotype-Tissue expression
IQR: Interquartile range
miR: Micro RNA
NBS: Newborn bloodspots
NEST: Newborn Epigenetic Study
OR: Odds ratio
PCR: Polymerase chain reaction
PSQ: Pyrosequencing
PTS: Prenatal tobacco smoke
SD: Standard deviation
