# Supplementary material for: Self-reported prenatal tobacco smoke exposure, AXL gene-body methylation, and childhood asthma phenotypes
Source: Clin Epigenetics. 2018 Jul 20;10:98. doi: 10.1186/s13148-018-0532-x (PMC6054742; doi:10.1186/s13148-018-0532-x)
Supplement: Supplementary file 1 — Supplemental methods. Table S1. Summary statistics for miR-199a1 and AXL mRNA expression, cotinine concentration and AXL methylation levels in CHS and NEST subjects. Table S2. Association between PTS exposure and AXL DNA methylation at each CpG site at birth in CHS and NEST subjects. Table S3. Association between plasma cotinine level and AXL methylation in NEST subjects. Table S4. Association between AXL mRNA expression and AXL methylation, PTS exposure and miR-199a1 expression in NEST subjects. Table S5. Association between miR-199a1 expression and AXL methylation and PTS exposure in NEST subjects. Table S6. Interaction between AXL DNA methylation at birth and PTS exposure in relation to risk of asthma and related symptoms at age 10 years in CHS subjects. Figure S1. Standard curve for testing PCR bias in Pyrosequencing assays. Figure S2. Plots for association between plasma cotinine level and AXL methylation using piecewise linear regression model. Figure S3. AXL mRNA expression levels across different tissues from the Genotype-Tissue expression (GTEx) project. (DOCX 790 kb) [file 13148_2018_532_MOESM1_ESM.docx]

**Self-reported prenatal tobacco smoke exposure, *AXL* gene-body methylation and childhood asthma phenotypes**

Lu Gao, Xiaochen Liu, Joshua Millstein, Kimberly D. Siegmund, Louis Dubeau, Rachel L. Maguire, Junfeng (Jim) Zhang, Bernard F. Fuemmeler, Scott H. Kollins, Cathrine Hoyo, Susan K. Murphy, Carrie V. Breton

**Additional File 1**

**Supplemental methods**

*mRNA expression in NEST*

Total *AXL* mRNA was isolated from stored PAXgene tubes of cord blood using the PAXgene blood miRNA isolation kit (Qiagen, Valencia, CA). First strand cDNA conversion from 1 µg mRNA was performed using the Origene’s cDNA synthesis kit (#HP100042). The Origene system’s approach takes into consideration that miRNAs are poor targets in qPCR detection due to their small sizes (21-23 nt) and to the lack of a poly A tail at the 3’ end, a feature that is essential in oligo dT primed cDNA synthesis. To overcome these obstacles, a protocol to add poly A tail to each miRNA was adopted, followed by the use of an anchor linker oligo dT to synthesize miRNA cDNA. Using 3 µl of cDNA stock (25 ng/µl), qPCR reactions were run with Kappa Sybr Fast qPCR kit (# KK4604; KapaBiosystems, Boston, MA) in the ABI 7900HT thermocycler (Thermofisher).

Origene’s qStar mRNA detection system (Origene, Rockville, MD) was used in the quantification of *AXL* mRNA in cord blood in NEST subjects. qPCR primers for the major *AXL* transcript (#HK228780) and its corresponding copy number standard (#HK201002) were designed by qStar. *AXL* mRNA was isolated from stored PAXgene tubes of cord blood using the PAXgene blood mRNA isolation kit (Qiagen, Valencia, CA). First strand cDNA conversion of mRNA was performed using Origene’s cDNA synthesis kit (#NP100042). qPCR reactions were run with Kappa Sybr Fast qPCR kit (# KK4604; KapaBiosystems, Boston, MA) in the ABI 7900HT thermocycler (Thermofisher). To evaluate reproducibility, 10% repeats were included.

| **Table S1. Summary statistics for miR-199a1 and *AXL* mRNA expression, cotinine concentration and *AXL* methylation levels in CHS and NEST subjects** | | | | | | | | | | | | | | | |
| --- | --- | --- | --- | --- | --- | --- | --- | --- | --- | --- | --- | --- | --- | --- | --- |
|  |  |  | CHS | | | | | |  | NEST | | | | | |
|  | Distance from TSS | Genomic region | N | Min | Mean ± SD | Median | IQR | Max |  | N | Min | Mean ± SD | Median | IQR | Max |
| MiR-199a1 expression (copies/µg RNA) |  |  |  |  |  |  |  |  |  | 222 | 200 | 257192 ± 225369 | 184098 | 258974 | 1187455 |
| *AXL* mRNA expression in cord blood (copies/µg cDNA) |  |  |  |  |  |  |  |  |  | 237 | 708 | 4479 ± 7279 | 3503 | 1965 | 96141 |
| Cotinine concentration (ng/ml) |  |  |  |  |  |  |  |  |  | 242 | 0.0 | 32.8 ± 66.5 | 1.0 | 28.0 | 371.0 |
| *AXL* methylation levels (%) |  |  |  |  |  |  |  |  |  |  |  |  |  |  |  |
| *Region 1* |  |  |  |  |  |  |  |  |  |  |  |  |  |  |  |
| CpG 25 | -508 | 5-Upstream | 799 | 0.0 | 43.0 ± 8.0 | 43.2 | 10.4 | 66.9 |  | 586 | 0.0 | 43.6 ± 9.8 | 44.3 | 11.9 | 79.7 |
| CpG 24 | -492 | 5-Upstream | 799 | 18.1 | 50.1 ± 7.5 | 50.4 | 10.2 | 67.8 |  | 586 | 1.8 | 51.2 ± 8.7 | 51.3 | 10.8 | 83.5 |
| CpG 23 | -479 | 5-Upstream | 799 | 0.0 | 20.1 ± 4.4 | 20.1 | 5.7 | 34.8 |  | 586 | 0.0 | 20.9 ± 5.4 | 21.1 | 5.8 | 44.3 |
| Average of CpG 23-25 |  |  | 799 | 13.4 | 37.7 ± 6.2 | 38.0 | 8.2 | 54.1 |  | 586 | 1.2 | 38.5 ± 7.1 | 39.0 | 8.9 | 60.6 |
| *Region 2* |  |  |  |  |  |  |  |  |  |  |  |  |  |  |  |
| CpG 9 | -55 | 5-Upstream | 797 | 0.0 | 11.7 ± 3.7 | 11.3 | 4.8 | 24.9 |  | 585 | 0.0 | 12.6 ± 4.9 | 12.4 | 5.2 | 61.3 |
| CpG 8 | -31 | 5-Upstream | 797 | 0.0 | 11.2 ± 3.3 | 10.9 | 4.1 | 24.1 |  | 585 | 0.0 | 13.0 ± 5.4 | 12.5 | 4.9 | 82.0 |
| Average of CpG 8-9 |  |  | 797 | 0.0 | 11.4 ± 3.4 | 11.1 | 4.2 | 23.5 |  | 585 | 0.0 | 12.8 ± 4.6 | 12.5 | 4.8 | 59.6 |
| *Region 3* |  |  |  |  |  |  |  |  |  |  |  |  |  |  |  |
| CpG 111 | 4518 | Intron 4 | 797 | 9.7 | 36.6 ± 3.6 | 36.5 | 4.4 | 51.6 |  | 583 | 0.0 | 37.6 ± 6.8 | 37.3 | 5.6 | 97.2 |
| *Region 4* |  |  |  |  |  |  |  |  |  |  |  |  |  |  |  |
| CpG 112 | 4550 | Intron 4 | 797 | 71.0 | 83.8 ± 3.1 | 84.1 | 4.0 | 92.4 |  | 583 | 4.3 | 82.8 ± 5.3 | 83.3 | 5.1 | 99.9 |
| *Region 5* |  |  |  |  |  |  |  |  |  |  |  |  |  |  |  |
| CpG 160 | 6813 | Intron 4 | 765 | 0.0 | 2.4 ± 1.0 | 2.3 | 1.1 | 6.7 |  | 564 | 0.0 | 2.6 ± 2.0 | 2.5 | 1.3 | 26.5 |
| CpG 161 | 6827 | Intron 4 | 798 | 0.0 | 4.6 ± 1.8 | 4.4 | 2.1 | 14.9 |  | 591 | 0.0 | 4.8 ± 2.2 | 4.6 | 2.3 | 28.5 |
| CpG 162 | 6834 | Intron 4 | 798 | 0.0 | 5.9 ± 2.1 | 5.7 | 2.7 | 16.0 |  | 591 | 0.0 | 6.4 ± 3.1 | 6.1 | 2.9 | 41.7 |
| Average of CpG 160-162 |  |  | 765 | 0.0 | 4.3 ± 1.5 | 4.1 | 1.8 | 10.3 |  | 564 | 0.0 | 4.6 ± 2.0 | 4.4 | 1.9 | 27.2 |
| *Region 6* |  |  |  |  |  |  |  |  |  |  |  |  |  |  |  |
| CpG 754 | 42562 | 3-UTR | 798 | 34.2 | 58.5 ± 3.5 | 58.6 | 4.2 | 69.0 |  | 587 | 6.5 | 56.1 ± 7.3 | 57.0 | 6.2 | 97.3 |
| TSS: transcription start site; UTR: untranslated region. | | | | | | | | | | | | | | | |

| **Table S2. Association between PTS exposure and *AXL* DNA methylation at each CpG site at birth in CHS and NEST subjects^a^** | | | | | | |
| --- | --- | --- | --- | --- | --- | --- |
|  | CHS (N=799) | | NEST (N=592) | | Pooled analysis (N=1294)^b^ | |
|  | 𝛽 (95% CI) | P-value | 𝛽 (95% CI) | P-value | 𝛽 (95% CI) | P-value |
| **Region 1** |  |  |  |  |  |  |
| CpG 25 | -0.77 (-2.15, 0.61) | 0.27 | 0.42 (-1.32, 2.16) | 0.64 | -0.38 (-1.46, 0.70) | 0.49 |
| CpG 24 | -0.99 (-2.28, 0.30) | 0.13 | -0.33 (-1.90, 1.24) | 0.68 | -0.78 (-1.77, 0.21) | 0.12 |
| CpG 23 | 0.30 (-0.46, 1.06) | 0.44 | 0.23 (-0.74, 1.21) | 0.64 | 0.27 (-0.34, 0.87) | 0.39 |
| **Region 2** |  |  |  |  |  |  |
| CpG 9 | 0.02 (-0.62, 0.66) | 0.95 | 0.18 (-0.71, 1.06) | 0.70 | 0.06 (-0.47, 0.59) | 0.82 |
| CpG 8 | 0.00 (-0.56, 0.57) | 1.00 | 1.01 (0.04, 1.98) | 0.04 | 0.41 (-0.07, 0.88) | 0.10 |
| **Region 3** |  |  |  |  |  |  |
| CpG 111 | 0.58 (-0.06, 1.22) | 0.08 | -0.22 (-1.48, 1.04) | 0.73 | 0.24 (-0.40, 0.88) | 0.47 |
| **Region 4** |  |  |  |  |  |  |
| CpG 112 | -0.12 (-0.67, 0.43) | 0.67 | 0.55 (-0.45, 1.54) | 0.28 | 0.14 (-0.41, 0.69) | 0.61 |
| **Region 5** |  |  |  |  |  |  |
| CpG 160 | 0.31 (0.13, 0.50) | 8.43E-04 | 0.26 (-0.13, 0.65) | 0.19 | 0.34 (0.13, 0.54) | 1.47E-03 |
| CpG 161 | 0.44 (0.13, 0.75) | 0.005 | 0.11 (-0.30, 0.53) | 0.60 | 0.32 (0.06, 0.58) | 0.01 |
| CpG 162 | 0.76 (0.40, 1.11) | 3.22E-05 | 0.56 (-0.01, 1.14) | 0.05 | 0.76 (0.43, 1.08) | 5.86E-06 |
| **Region 6** |  |  |  |  |  |  |
| CpG 754 | 0.46 (-0.16, 1.09) | 0.14 | -0.53 (-1.88, 0.81) | 0.44 | -0.10 (-0.78, 0.57) | 0.77 |
| ^a^ Adjusted for child's sex, ethnicity (of child in CHS and of mother in NEST), gestational age, maternal age at delivery, and parental education level | | | | | | |
| ^b^ Pooled analysis only included black, Hispanic and non-Hispanic white subjects | | | | | | |
| Estimates are showing percent changes in methylation. | | | | | | |

| **Table S3. Association between plasma cotinine level and *AXL* methylation in NEST subjects (N=242)^a^** | | | | | |
| --- | --- | --- | --- | --- | --- |
|  | Nonsmoker | Passive smoker | | Active smoker | |
|  |  | 𝛽 (95% CI) | P-value | 𝛽 (95% CI) | P-value |
| Region 1 | Ref | 1.94 (-0.34, 4.22) | 0.09 | 1.06 (-1.07, 3.19) | 0.33 |
| Region 2 | Ref | 0.71 (-0.67, 2.08) | 0.31 | -0.004(-1.29, 1.28) | 0.99 |
| Region 3 | Ref | 0.70 (-1.31, 2.71) | 0.49 | 0.54 (-1.34, 2.42) | 0.57 |
| Region 4 | Ref | 0.94 (-1.44, 3.32) | 0.44 | 1.08 (-1.14, 3.31) | 0.34 |
| Region 5 | Ref | 0.16 (-0.55, 0.87) | 0.65 | 0.17 (-0.49, 0.83) | 0.61 |
| Region 6 | Ref | -1.37 (-3.46, 0.72) | 0.20 | 0.99 (-0.96, 2.94) | 0.32 |
| ^a^ Adjusted for child's sex, maternal ethnicity, gestational age, maternal age at delivery, parental education level and maternal BMI during pregnancy. Cotinine was categorized as nonsmoker (0-1 ng/ml, N=119), passive smoker (1-10 ng/ml, N=47), and active smoker (>10 ng/ml, N=76). Estimates are showing percent changes in methylation. | | | | | |

| **Table S4. Association between *AXL* mRNA expression and *AXL* methylation, PTS exposure and miR-199a1 expression in NEST subjects (N=236)^a^** | | |
| --- | --- | --- |
|  | Percent change in *AXL* mRNA (95% CI) | P-value |
| *AXL methylation* |  |  |
| Region 1 | 0.4 (-0.7, 1.6) | 0.46 |
| Region 2 | 1.5 (-0.3, 3.3) | 0.11 |
| Region 3 | -0.6 (-1.6, 0.3) | 0.20 |
| Region 4 | -1.0 (-2.1, 0.1) | 0.07 |
| Region 5 | 2.8 (-0.7, 6.5) | 0.12 |
| Region 6 | -0.9 (-2.0, 0.2) | 0.10 |
| *Maternal smoking* |  |  |
| Self-reported PTS | -7.4 (-22.5, 10.6) | 0.39 |
| Plasma cotinine level^b^ | 0.0 (-0.4, 0.4) | 0.91 |
| *microRNA^c^* |  |  |
| miR-199a1 | -0.8 (-1.8, 0.2) | 0.10 |
| ^a^ All models were adjust for child's sex, maternal ethnicity, gestational age, maternal age at delivery, maternal education level and maternal BMI during pregnancy; additionally adjusted for miRNA plate number for model with miR-199a1 | | |
| ᵇ N=98; results showing for per 10% increase in cotinine level | | |
| ^c^ N=202; results showing for per 10% increase in miR-199a1 level | | |

| **Table S5. Association between miR-199a1 expression and *AXL* methylation and PTS exposure in NEST subjects (N=210)^a^** | | |
| --- | --- | --- |
|  | Percent change in miR-199a1 (95% CI) | P-value |
| *AXL methylation* |  |  |
| Region 1 | 1.0 (-0.5, 2.5) | 0.21 |
| Region 2 | -0.3 (-3.0, 2.4) | 0.81 |
| Region 3 | 1.0 (-0.5, 2.4) | 0.20 |
| Region 4 | 2.1 (0.7, 3.5) | 0.004 |
| Region 5 | -2.2 (-8.8, 4.9) | 0.53 |
| Region 6 | 1.5 (-0.3, 3.4) | 0.10 |
| *Maternal smoking* |  |  |
| Self-reported PTS | -21.2 (-37.9, -0.1) | 0.05 |
| Plasma cotinine level^b^ | -0.8 (-1.3, -0.2) | 0.006 |
| ᵃ All models were adjusted for miRNA plate number, child's sex, maternal ethnicity, gestational age, maternal age at delivery, maternal education level and maternal BMI during pregnancy | | |
| ^b^ N=90; results showing for per 10% increase in cotinine level | | |

| **Table S6. Interaction between *AXL* DNA methylation at birth and PTS exposure in relation to risk of asthma and related symptoms at age 10 years in CHS subjects (N=799)^a^** | | | | | | | | | | | | | | | | | |
| --- | --- | --- | --- | --- | --- | --- | --- | --- | --- | --- | --- | --- | --- | --- | --- | --- | --- |
|  | **Ever MD-diagnosed asthma** | | | | |  | **Ever wheezing** | | | | |  | **Wheezing in the previous 12 months** | | | | |
|  | Unexposed to PTS | | Exposed to PTS | | Interaction P-value |  | Unexposed to PTS | | Exposed to PTS | | Interaction P-value |  | Unexposed to PTS | | Exposed to PTS | | Interaction P-value |
|  | OR   (95% CI) | P-value | OR   (95% CI) | P-value |  |  | OR   (95% CI) | P-value | OR   (95% CI) | P-value |  |  | OR   (95% CI) | P-value | OR   (95% CI) | P-value |  |
| Region 1 | 0.75  (0.48, 1.16) | 0.20 | 0.51  (0.24, 1.07) | 0.07 | 0.47 |  | 1.06  (0.73, 1.55) | 0.76 | 0.85   (0.49, 1.49) | 0.58 | 0.53 |  | 1.03  (0.65, 1.64) | 0.90 | 0.69  (0.36, 1.30) | 0.25 | 0.27 |
| Region 2 | 0.90  (0.58, 1.41) | 0.64 | 0.60  (0.27, 1.33) | 0.21 | 0.44 |  | 0.80  (0.54, 1.17) | 0.25 | 0.94   (0.53, 1.66) | 0.83 | 0.63 |  | 1.09  (0.69, 1.73) | 0.71 | 0.79  (0.40, 1.54) | 0.49 | 0.46 |
| Region 3 | 0.75  (0.49, 1.16) | 0.20 | 0.64  (0.30, 1.35) | 0.24 | 0.82 |  | 0.68  (0.46, 0.99) | 0.04 | 1.12   (0.63, 1.99) | 0.69 | 0.19 |  | 0.95  (0.60, 1.51) | 0.82 | 1.11  (0.57, 2.19) | 0.75 | 0.61 |
| Region 4 | 0.76  (0.50, 1.16) | 0.21 | 1.18  (0.55, 2.55) | 0.67 | 0.40 |  | 0.87  (0.60, 1.26) | 0.47 | 1.32   (0.72, 2.42) | 0.37 | 0.15 |  | 0.74  (0.47, 1.16) | 0.18 | 1.27  (0.63, 2.59) | 0.50 | 0.08 |
| Region 5 | 0.81  (0.51, 1.29) | 0.38 | 0.79  (0.37, 1.70) | 0.55 | 0.89 |  | 0.61  (0.41, 0.92) | 0.02 | 0.87   (0.49, 1.55) | 0.64 | 0.40 |  | 0.85  (0.53, 1.37) | 0.51 | 0.78   (0.40, 1.54) | 0.48 | 0.83 |
| Region 6 | 0.94  (0.62, 1.42) | 0.78 | 0.68  (0.30, 1.54) | 0.36 | 0.50 |  | 1.15  (0.79, 1.67) | 0.46 | 1.03  (0.56, 1.92) | 0.91 | 1.00 |  | 0.81  (0.52, 1.25) | 0.33 | 0.97   (0.48, 1.96) | 0.93 | 0.62 |
| ^a^ Adjusted for child's sex, ethnicity, and city of residence at study entry. Odds ratios are scaled to per 2SD change in methylation. | | | | | | | | | | | | | | | | | |

**Figures**

**
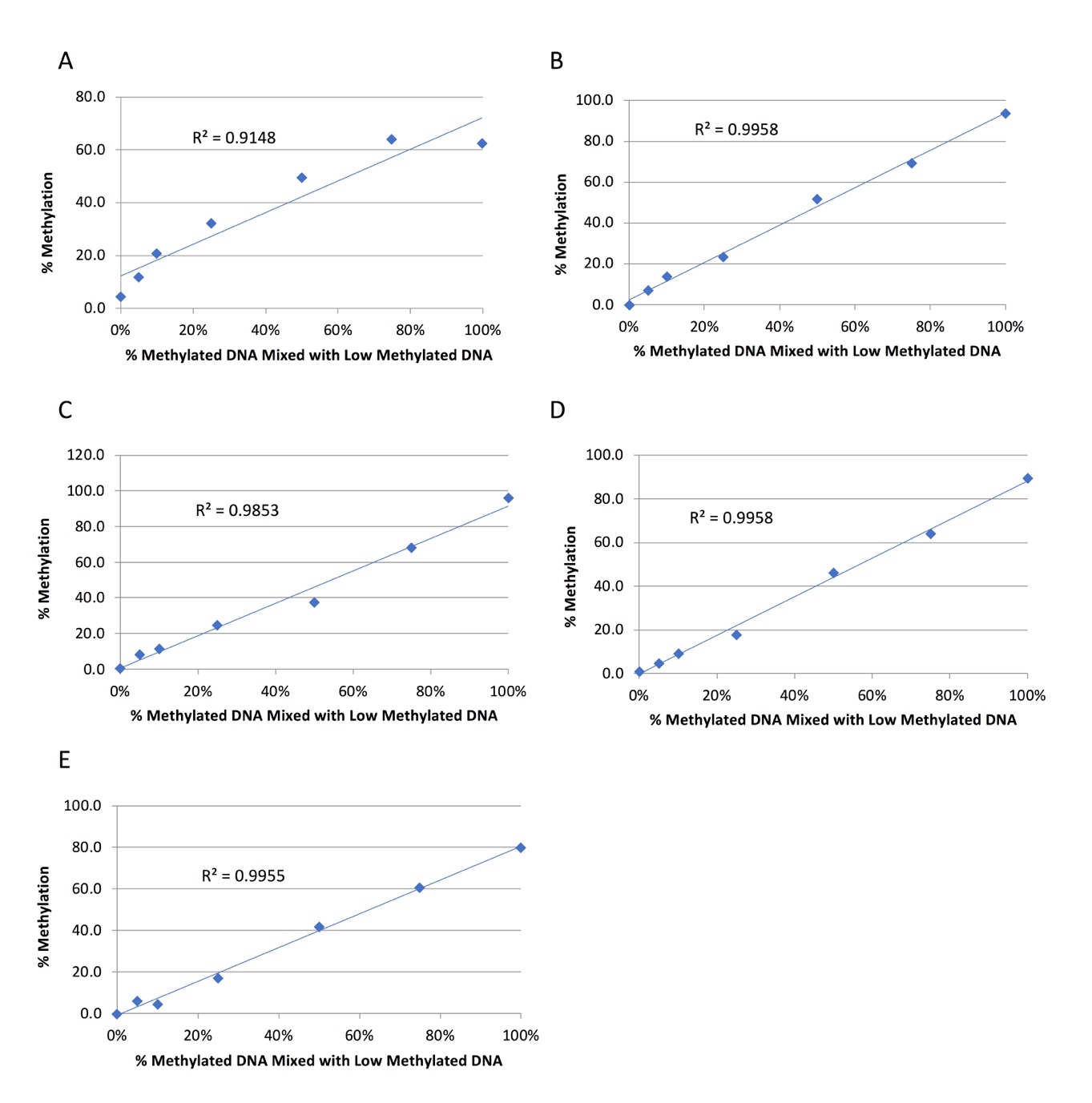
**

**Figure S1.** Standard curve for testing PCR bias in Pyrosequencing assays for Region 1 (panel A), Region 2 (panel B), Regions 3 and 4 (panel C), Region 5 (panel D), and Region 6 (panel E) (see methods for details). R-square for testing the linear trend was displayed.


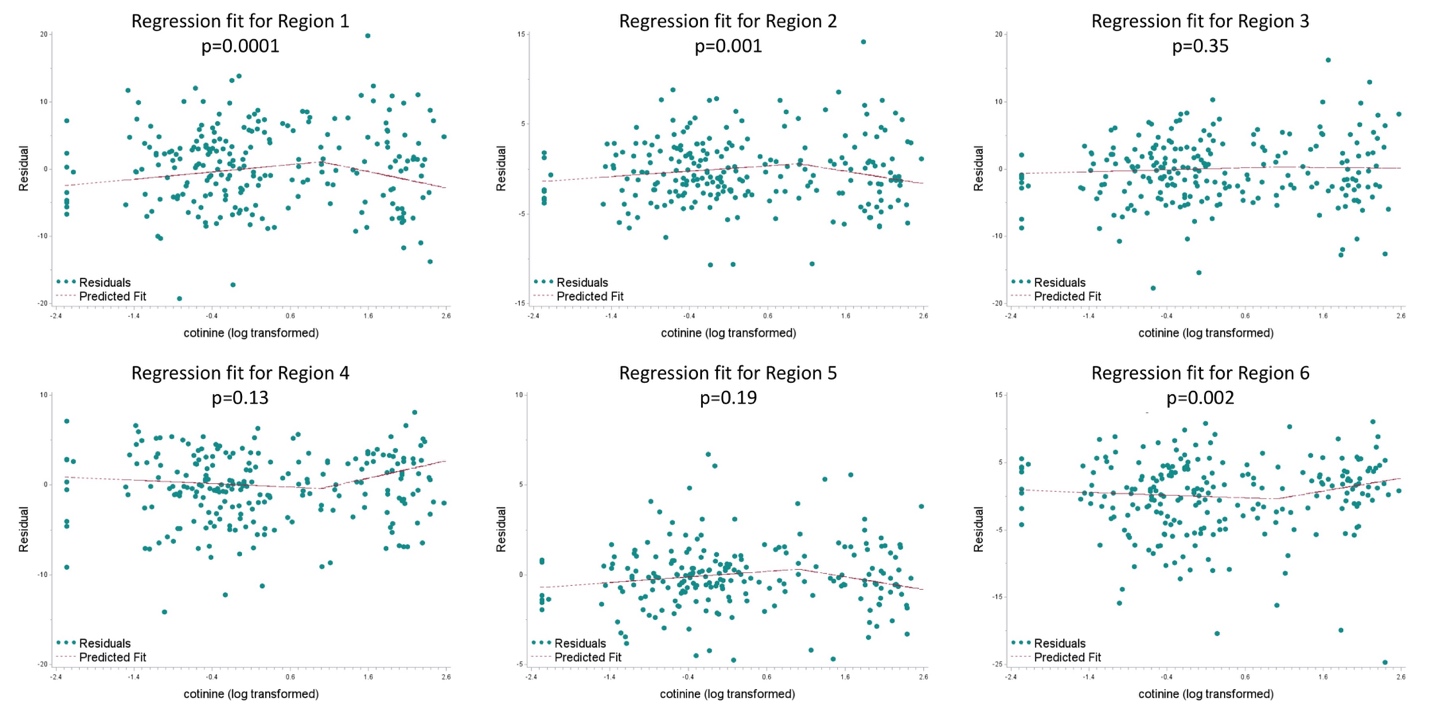


**Figure S2**. Plots for association between plasma cotinine level and *AXL* methylation using piecewise linear regression model (see statistical methods for details). P-values are showing the overall fit of the two-component linear spline. Cotinine was log10 transformed.

Green dots: residuals from model. Red line: fitted regression line for each component of spline.


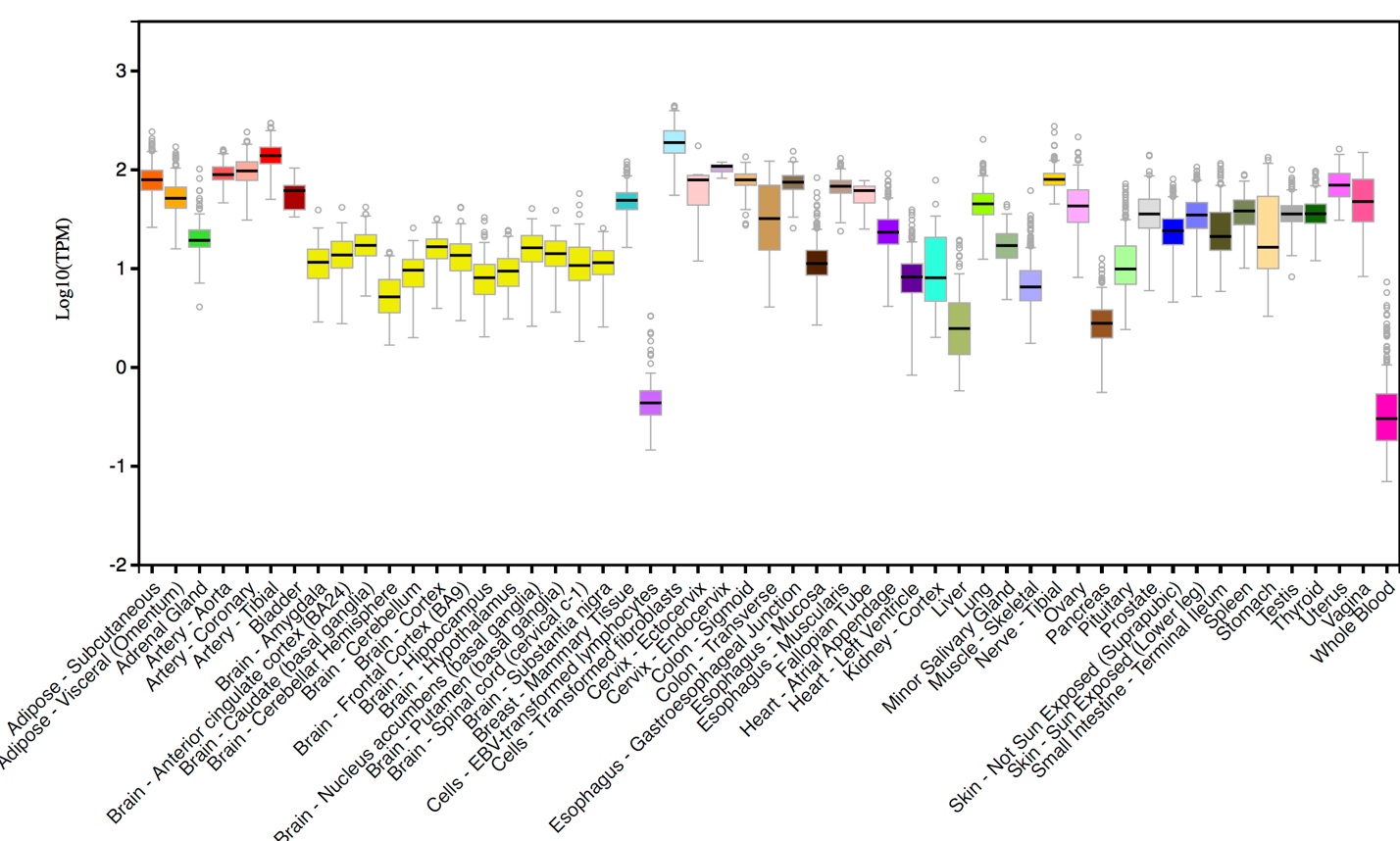


**Figure S3**. *AXL* (Gencode ID: ENSG00000167601.7) mRNA expression levels across different tissues from the Genotype-Tissue expression (GTEx) project.
